# The Covert Surge: Murine Bile Acid Levels Are Associated With Pruritus in Pediatric Autoimmune Sclerosing Cholangitis

**DOI:** 10.3389/fped.2022.903360

**Published:** 2022-05-11

**Authors:** Katharina Meinel, Doloresz Szabo, Antal Dezsofi, Sina Pohl, Tanja Strini, Theresa Greimel, Victor Aguiriano-Moser, Harald Haidl, Martin Wagner, Axel Schlagenhauf, Jörg Jahnel

**Affiliations:** ^1^Division of General Pediatrics, Department of Pediatrics and Adolescent Medicine, Medical University of Graz, Graz, Austria; ^2^First Department of Pediatrics, Semmelweis University, Budapest, Hungary; ^3^Division of Gastroenterology and Hepatology, Department of Internal Medicine, Medical University of Graz, Graz, Austria

**Keywords:** muricholic acid, autotaxin (ATX), bile acid (BA), autoimmune sclerosing cholangitis, pediatrics, pruritus, progressive familial intrahepatic cholestasis

## Abstract

**Objectives:**

The exact etiology of pruritus in chronic cholestasis is unknown. Pruritus intensity does not correlate with common biochemical indices and there is a lack of biomarkers guiding diagnosis and treatment. We explored profiles of bile acids (BA) and muricholic acids (MCA) as well as autotaxin (ATX) antigen levels as potential circulating biomarkers of pruritus in pediatric patients.

**Methods:**

In 27 pediatric cholestatic patients [autoimmune sclerosing cholangitis (ASC) *n* = 20 (with pruritus *n* = 6, without pruritus *n* = 14); progressive familial intrahepatic cholestasis (PFIC) *n* = 7 (with pruritus *n* = 5, without pruritus *n* = 2)] and 23 age-matched controls pruritus was assessed by a visual analog scale of pruritus (PVAS). We obtained profiles of serum human BA including MCA using a mass-spectrometry assay and ATX antigen levels with a commercial ELISA.

**Results:**

PFIC and ASC patients exhibited significantly higher BA-, and MCA levels, than healthy controls, but only PFIC patients showed elevated ATX antigen levels higher [median: 1,650 ng/ml, interquartile rang (IQR): 776.9–3,742] compared to controls (median: 315.9 ng/ml, IQR: 251.1–417.2; PFIC *p* = 0.0003). ASC patients with pruritus showed only a minor increase in total BA (tBA) levels (median: 76.5 μmol/L, IQR: 54.7–205), but strikingly higher T-conjugated BA (median: 16.4 μmol/L, IQR: 8.9–41.4) and total MCA (tMCA) (median: 1.15 μmol/L, IQR: 0.77–2.44) levels compared to ASC patients without pruritus (tBA median: 24.3 μmol/L, IQR: 16.2–80.8; *p* < 0.0408; T-conjugated BA median: 1.3 μmol/L, IQR: 0.8–4.9; *p* = 0.0023; tMCA median: 0.30 μmol/L, IQR: 0.13–0.64, *p* = 0.0033). BA/MCA profiles distinctly differed depending on presence/absence of pruritus. Different from PFIC patients, ATX antigen levels were not significantly elevated in ASC patients with (median: 665.8 ng/ml, IQR: 357.8–1,203) and without pruritus (median: 391.0 ng/ml, IQR: 283.2–485.6). In ASC patients, tBA, tMCA, and ATX antigen levels did not correlate with pruritus severity.

**Conclusion:**

Despite the same underlying disease, pediatric ASC patients with pruritus exhibit significantly altered BA profiles and MCA levels compared to ASC patients without pruritus. ATX antigen levels seem to have little diagnostic or prognostic meaning in ASC patients. An increased ATX activity alone seems not to be causal for pruritus genesis in ASC patients.

**Clinical Trial Registration:**

[www.drks.de], identifier [DRKS00026913].

## Introduction

Chronic cholestatic liver diseases (CCLD) are associated with high morbidity and mortality and represent the leading indication for liver transplantation in pediatric patients. Disorders of childhood presenting with chronic cholestasis include autoimmune sclerosing cholangitis (ASC), progressive familial intrahepatic cholestasis (PFIC), and biliary atresia amongst others (1, 2). In children, various CCLD are frequently associated with intractable chronic pruritus. However, pruritus is not imperative in these patients and the exact underlying pathogenesis is unknown (3–5). In the past, enhanced bile acid (BA) levels have been implicated in the etiology of cholestatic pruritus (6, 7). However, no correlation between itch severity and BA levels has ever been established (6, 7) and an occasional reduction of pruritus has been reported in some patients despite progressing cholestatic disorders and persistently increased BA levels (6–8).

Primary BA are synthesized from cholesterol in the liver whereas secondary BA are formed by modification of primary BA in the distal intestine and the colon by bacterial enzymes (9–11). In humans, the primary BA chenodeoxycholic acid (CDCA) and cholic acid (CA) are predominant (9, 12, 13), whereas in mice, tri-hydroxylated, so-called muricholic bile acids (MCA), are prevailing (14). However, MCA were found to be also synthesized in neonates and patients with CCLD (14, 15). Under physiological conditions the major constituent of BA undergoes enterohepatic circulation between liver and small intestine (16, 17), a process which is mainly regulated by BA themselves via the nuclear receptor farnesoid X (FXR) (11, 18, 19). The activation of FXR leads to downregulation of BA uptake systems like ileal BA transporter (IBAT) and increased expression of exporter proteins such as multidrug resistance associated protein 2 and 3 (MRP2/MRP3) amongst others in liver as well as in intestine (11, 16, 20, 21). To increase hydrophilicity most of the BA, but also MCA, are conjugated with either glycine (G) or taurine (T) (10, 22). Two independent enzymatic reactions are particularly involved in the conjugation of BA in the human liver: BA-CoA synthetase (BACS) and BA-CoA amino acid N-acetyltransferase (BAT) (23, 24). The genes encoding for these two key enzymes are direct targets of FXR (24). This may be a hepatoprotective effect as conjugated BA are considered less toxic. Table 1 gives an overview of all BA/MCA investigated in this study.

**TABLE 1:** Unconjugated bile acids (BA) and C-6 hydroxylated muricholic bile acids (MCA) and their glycine (G) or taurine (T) conjugates analyzed in this study design.

Unconjugated BAs	G-conjugated BA	T-conjugated BA
Cholic acid (CA)*	GCA	TCA
Chenodeoxycholic acid (CDCA)*	GCDCA	TCDCA
Deoxycholic acid (DCA)	GDCA	TDCA
Lithocholic acid (LCA)	GLCA	TLCA
Ursodeoxycholic acid (UDCA)	GUDCA	TUDCA
α -muricholic acid (AMCA)**	GAMCA	TAMCA
β -muricholic acid (BMCA)**	GBMCA	TBMCA
γ-muricholic acid (GMCA)	GGMCA	TGMCA
ω-muricholic acid (OMCA)	GOMCA	TOMCA
Hyodeoxycholic acid (HDCA)	GHDCA	THDCA

*Primary BA; **Primary MCA.

Besides BA, enhanced levels of lysophosphatidic acid (LPA), a potent neuronal activator generated by the enzyme autotaxin (ATX), are assumed to be involved in the pathophysiology of cholestatic pruritus (25, 26). A substantial increase of LPA was found in patients with chronic cholestatic pruritus compared to patients without pruritus (26). Moreover, ATX activity correlated with itch intensity and total BA (tBA) levels in adult and pediatric patients (26, 27), however, the underlying reason for the ATX surge has not been found yet.

We investigated human BA and MCA in pediatric ASC and PFIC patients to test whether specific patterns of human BA including MCA allow discrimination between pediatric ASC/PFIC patients with and without pruritus based solely on laboratory findings that could be used as biomarkers in the future. Furthermore, we wanted to investigate how these alterations correlate with ATX levels and severity of pruritus to gain insight into their specific contribution.

## Materials and Methods

### Study Design and Patients’ Characteristics

We conducted a prospective study at the First Department of Pediatrics of the Semmelweis University Hungary and the Department of Pediatrics and Adolescents Medicine of the Medical University of Graz, for which we collected serum samples of 50 children aged 1–18 years between August 2018 and March 2020. We included 20 children with ASC (with pruritus *n* = 6, without pruritus *n* = 14) and 7 children with PFIC [with pruritus *n* = 5 (PFIC II *n* = 5), without pruritus *n* = 2 (PFIC I *n* = 1, PFIC II *n* = 1)], and 23 healthy age-matched controls (Supplementary Figure 1). ASC and PFIC were diagnosed according to the guidelines (28) by liver biopsy and biliary imaging under consideration of laboratory changes of biomarkers indicating hepatobiliary injury. None of our included patients had liver cirrhosis. After an overall comparison of ASC, PFIC patients, and the control group, only ASC patients were stratified according to presence/absence of pruritus. The PFIC cohort had an insufficient number of patients without pruritus.

### Visual Analog Scale of Pruritus

The PVAS represents the numbers 0 (“no itch”) to 10 (“worst imaginable itch”). Patients are asked to rate the intensity of their itching using this scale. It features high reliability and concurrent validity and is a popular choice for children 5 years of age, due to its simple format (29). In younger children (<5 years of age), we differentiated only by the existence of pruritus (pruritus: “yes” or “no”). Patients with a PVAS value of 0 were included in the study group of patients without pruritus.

### Blood Samples

Non-fasting blood sampling was performed during the routine diagnostic workup. Venous blood samples were collected in serum tubes. Serum of all patients for determination of BA/MCA concentrations and composition of the BA/MCA pool and ATX activity was obtained by centrifugation (2,000 × g, 10 min) before it was frozen at −80°C within 2 h and stored until analysis.

### Laboratory Analysis

Concentrations of bilirubin, alanine aminotransferase (ALT), aspartate aminotransferase (AST), gamma-glutamyl transpeptidase (GGT), alkaline phosphatase (AP) and lactate dehydrogenase (LDH) were measured by standard laboratory methods.

### Bile Acid Analysis

BA/MCA levels including unconjugated, T-, and G-conjugated BA/MCA species (Table 1) were measured by high-performance liquid chromatography (HPLC) combined with mass spectrometry (MS) as described previously (30). Briefly, plasma samples were prepared after the protocol of Humbert et al. (31). After addition of internal standards d4-DCA, d4-LCA, d4-GLCA, d4-GCDCA, and d4-TDCA, 0.2 nmol each, plasma samples (10 μl) were vortexed for 1 min. 400 μl of acetonitrile (80% v/v; Sigma Aldrich, Taufkirchen, Germany) were added for deproteination. After vortexing, the precipitate was removed by centrifugation at 3,200 g for 12 min. The supernatant was dried under a stream of nitrogen (40°C). The samples were re-dissolved in 100 μl of mobile phase B (methanol with 1.2% v/v formic acid and 0.38% w/v ammonium acetate) and transferred to an autosampler. Individual BA were separated by HPLC using a reversed-phase C18 column (Macherey-Nagel, Düren, Germany) and a kinetex pentafluorophenyl column (Phenomenex, Aschaffenburg, Germany). Quantification and characterization were achieved using a Q Exactive™ mass spectrometer (Thermo Fisher Scientific, Waltham, MA) and a high-performance quadrupole precursor selection with high-resolution and accurate-mass (HR/AM) Orbitrap™ detection (30).

### Human ENPP-2/Autotaxin Immunoassay

The quantitative determination of human ENPP-2/ATX- concentrations in serum samples was performed using the Quantikine^®^ ELISA Human ENPP-2-/ATX Immunoassay (Abcam, Cambridge, MA). Serum samples were diluted 20–50-fold before the assay according to protocol and results were multiplied by the respective dilution factor.

### Ethics

This clinical study has been approved by the Hungarian Ethics Committee (43477-/2018/EKU) and the Austrian Ethics Committee (31–337 ex 18/19) and was performed in accordance with the ethics standards as laid down in the 1964 Declaration of Helsinki and its later amendments or comparable ethical standards. Parental consent was obtained for each subject. The trial was registered at the German Clinical Trials Register.^1^ The trial registration number is DRKS00026913.

### Statistical Analysis

**FIGURE 1:**
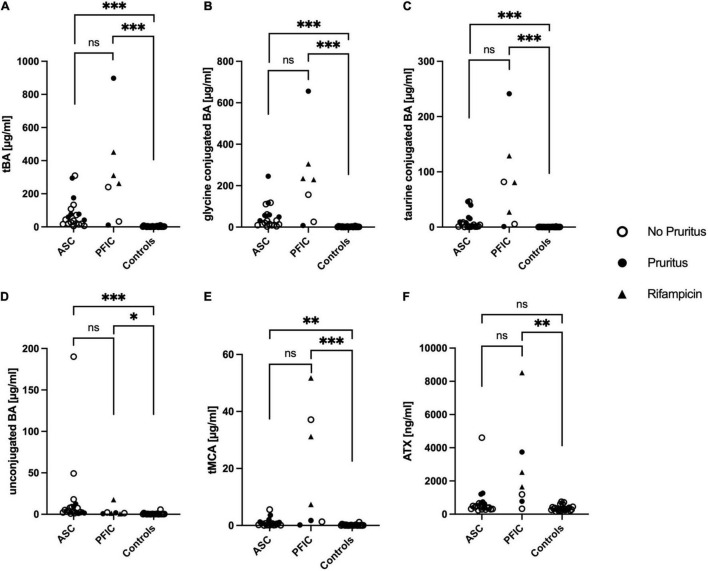
Total bile acids (tBA) **(A)**, taurine conjugated BA **(B)**, glycine conjugated BA **(C)**, unconjugated BA **(D)**, total muricholic bile acids (tMCA) **(E)**, and autotaxin (ATX) antigen levels **(F)** in pediatric patients with ASC, PFIC and healthy age-matched controls. Significances are indicated with bars and stars within the diagram. *p < 0.05; **p < 0.01; ***p < 0.001.

**FIGURE 2:**
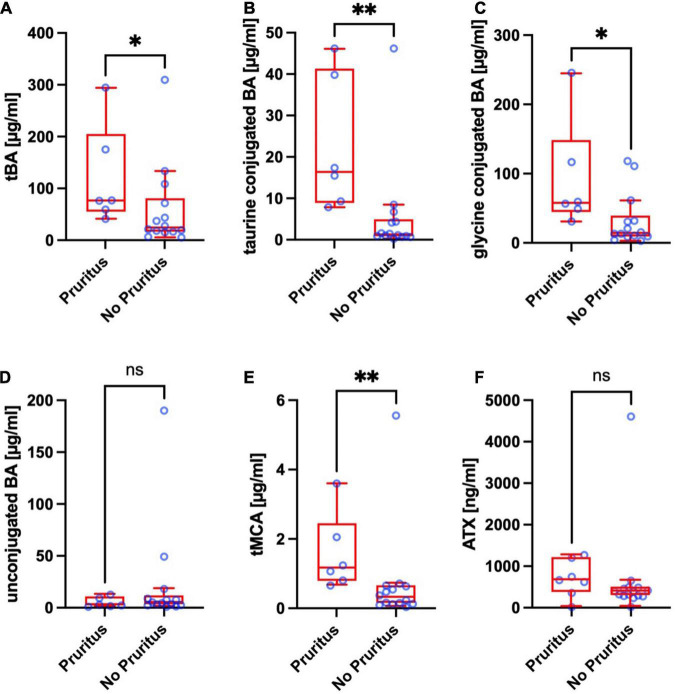
Total bile acids (tBA) **(A)**, taurine conjugated BA **(B)**, glycine conjugated BA **(C)**, unconjugated BA **(D)**, total muricholic bile acids (tMCA) **(E)**, and autotaxin (ATX) antigen levels **(F)** of pediatric ASC patients with and without pruritus. Significances are indicated with bars and stars within the diagram. *p < 0.05; **p < 0.01.

**FIGURE 3:**
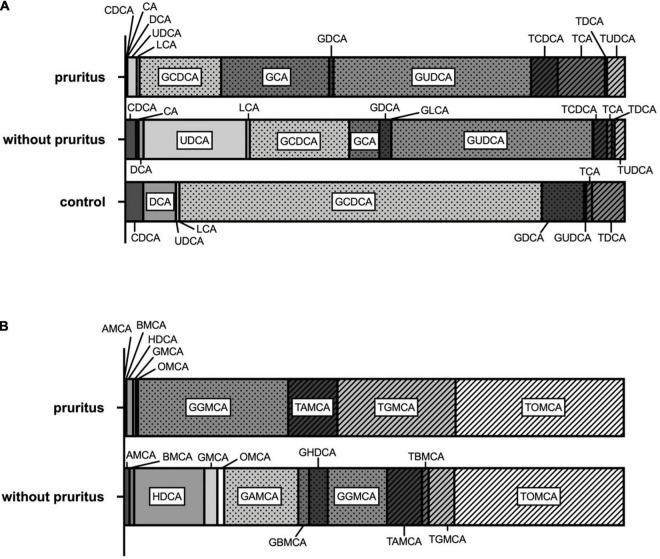
Bile acid profiles **(A)** and muricholic acid profiles **(B)** in ASC patients with and without pruritus.

The statistical analysis was based on clinical and laboratory findings. A Kruskal-Wallis analysis followed by Dunn’s multiple comparison test was performed to compare data of ASC patients, PFIC patients, and the control group. Comparison of data from ASC patients with/without pruritus was done via Man-Whitney *U*-test. We calculated BA/MCA profiles for all our ASC patients with and without pruritus and healthy age-matched controls. BA profiling was done through determination of the proportion of every single BA when expressed as a percentage of the tBA level. Spearman’s correlation coefficient and corresponding *p*-values were used to investigate the relationships between the examined parameters. All statistical analysis was performed in GraphPad Prism software. The changes in the examined parameters (Figures 1–3) were visualized using GraphPad Prism Software.

## Results

### Demographic Data and Clinical Characteristics

The 7 included patients with PFIC were significantly younger [median age 1.5 years, interquartile range (IQR): 1.2–3.0] compared to ASC patients (median age 16 years, IQR: 15–17.8, *p* < 0.0001) and controls (median age 14 years, IQR: 12.0–16.0, *p* = 0.0279). This is also reflecting in the smaller height and weight of PFIC patients (median height: 86.0 cm, IQR: 83.0–101.0, median weight: 12.3 kg, IQR: 11.1–16.1) compared to ASC patients (median height 164.0 cm, IQR: 156.3–173.8, *p* = 0.0004; median weight: 56.1 kg, IQR: 46.9–67.1, *p* = 0.0001) and controls (median height: 165.0 cm, IQR: 155.0–173.0, *p* = 0.0010; median weight 57.0 kg, IQR: 42.0–65.0, *p* 0.0002). All patients with PFIC received oral ursodeoxycholic acid (UDCA). Children with PFIC and intractable pruritus were treated with rifampicin as additional medication (*n* = 4).

The baseline demographic and clinical characteristics of pediatric ASC patients with and without pruritus and healthy age-matched controls are summarized in Table 2. ASC patients with and without pruritus did not differ significantly concerning age, height, and weight. ASC patients with pruritus showed significantly higher ALT levels (median: 85.5 U/l, IQR: 42.3–135.8) and AST levels (median: 84.5 U/l, IQR: 45.8–84.5) compared to ASC patients without pruritus (ALT: median: 28.5 U/l, IQR: 14.3–59.3, *p* = 0.0489; AST: median: 32.0 U/l, IQR: 27.3–48.5, *p* = 0.0322). Furthermore, ASC patients with pruritus had significantly higher serum bilirubin levels (median: 26.0 μmol/l, IQR: 13.5–79.0) than ASC patients without pruritus (median: 10.0 μmol/l, IQR: 6.5–17.9, *p* = 0.0049). 5 out of 6 patients with ASC with pruritus received oral UDCA. The mean daily dose of UDCA in our ASC patients with and without pruritus is listed in Table 2. In both, pediatric ASC patients with and without pruritus, inflammatory bowel disease (IBD) was the most prevalent comorbidity (IBD in ASC with pruritus *n* = 3; IBD in ASC without pruritus *n* = 5). The affected patients received additional medication such as azathioprine, mesalamine, prednisolone, or tacrolimus (Table 2).

**TABLE 2:** Demographic data and biochemical characteristics of ASC patients with and without pruritus and controls.

	ASC pruritus (*n* = 6)	ASC without pruritus (*n* = 14)	*P*-value*	Controls (*n* = 23)	*P*-value**
**Age (years)**	15 (15.0–17.3)	16 (13.8–18.0)	0.8228	14 (12.0–16.0)	0.1907
**Sex, female, *n* (%)**	4 (53)	5 (31)	0.3359	10 (43)	0.3898
**Height (cm)**	159.0 (148.1–177.8)	166.0 (158.9–174.3)	0.4324	165 (155.0–173.9)	0.8852
**Weight (kg)**	54.9 (50.0–60.7)	56.7 (46.6–68.4)	0.9044	57.0 (42.0–65.0)	0.7634
**Comorbidities, *n*:**			0.2865	0	–
IBD	3	5			
Diabetes mellitus I	1	–			
Protein S deficiency	1	–			
**PVAS**	5 1.9	0	**<0.0001**	0	**<0.0001**
**Serum ALP (U/l)**	343.0 (227.3–437.3)	192.0 (112.0–212.5)	0.0549	78.0 (53.0–141.0)	**<0.0001**
**Serum GGT (U/l)**	126.5 (50.5–285.3)	50.5 (26.8–109.3)	0.0507	16.0 (14.0–16.0)	**<0.0001**
**Serum ALT (U/l)**	85.5 (42.3–135.8)	28.5 (14.3–59.3)	**0.0489**	17 (13.0–21.0)	**<0.0001**
**Serum AST (U/l)**	84.5 (45.8–136.8)	32.0 (27.3–48.5)	**0.0322**	24.0 (19.0–27.0)	**<0.0001**
**LDH (U/l)**	211.0 (175.0–248.0)	170.0 (148.3–198.5)	0.1093	189.0 (157.0–226.0)	0.3086
**Total serum bilirubin (μ mol/l)**	26.0 (13.5–79.0)	10.0 (6.5–17.0)	**0.0049**	0.4 (0.3–0.6)	**< 0.0001**
**UDCA, n**	5	11	>0.9999	–	–
**Dosage 10 mg/kg/d**	500	500	0.8404	–	–
**(Maximum 500 mg)**					
**Additional medication, *n*:**			0.6717	–	–
Azathioprine	5	11			
Mesalamine (5-ASA)	2	4			
Prednisolone	1	3			
Furosemide	1	0			
Spironolactone	1	1			

*After the median values, interquartile ranges are shown in brackets.*

*Compared to ASC patients with pruritus. Significant differences are shown in bold. **Compared to ASC patients with pruritus. Significant differences are shown in bold.

*ALP, alkaline phosphatase; ALT, alanine aminotransferase; ASC, autoimmune sclerosing cholangitis; AST, aspartate aminotransferase; GGT, gamma-glutamyl transferase; IBD, inflammatory bowel disease; LDH, lactate dehydrogenase; PVAS, pruritus visual analog scale; UDCA, ursodeoxycholic acid.*

### Comparison of Bile Acid-, Muricholic Acid- and Autotaxin Antigen Levels Between Pediatric Autoimmune Sclerosing Cholangitis Patients, Progressive Familial Intrahepatic Cholestasis Patients, and Healthy Controls

TBA levels were significantly elevated in both, ASC patients (median: 42.4 μmol/L, IQR: 18.5–100.1) and PFIC patients (median: 262.6 μmol/L, IQR: 32.3–451.7) compared to age-matched controls (median: 1.7 μmol/L, IQR: 0.9–3.6; ASC *p* < 0.0001; PFIC *p* < 0.0001). There was no statistically significant difference in tBA levels between ASC and PFIC patients (*p* = 0.7936) (Figure 1A). The G- and T-conjugated BA, as well as the unconjugated BA, were significantly higher in ASC- and PFIC patients compared to age-matched controls (ASC: G-conjugated BA *p* < 0.0001, T-conjugated BA *p* < 0.0001, unconjugated BA *p* < 0.0001; PFIC: G-conjugated BA *p* < 0.0001, T-conjugated BA *p* < 0.0001, unconjugated BA *p* = 0.0262) (Figures 1B–D and Table 3). However, no statistically significant difference between ASC and PFIC patients concerning G-conjugated BA (*p* = 0.6911), T-conjugated BA (*p* = 0.4733), or unconjugated BA (*p* = 0.5628) could have been found (Figures 1B–D and Table 3).

**TABLE 3:** Total bile acid levels in pediatric ASC- and PFIC patients with and without pruritus and controls.

Bile acid (BA) (μ mol/L)	ASC (*n* = 20) median (IQR)	PFIC (*n* = 16) median (IQR)	*P*-value*	Controls (*n* = 23) median (IQR)	*P*-value**	*P*-value***
Total BA	42.4 (18.5–100.1)	262.6 (32.3–451.7)	0.7936	1.7 (0.9–3.6)	**<0.0001**	**<0.0001**
G-conjugated BA	30.8 (11.0–60.8)	229.2 (25.3–305.1)	0.6911	1.4 (0.5–2.7)	**<0.0001**	**<0.0001**
T-conjugated BA	4.3 (0.9–13.9)	80.7 (5.5–129.0)	0.4733	0.2 (0.0–0.3)	**<0.0001**	**<0.0001**
Unconjugated BA	3.9 (2.3–9.2)	3.9 (2.3–9.2)	0.5628	0.3 (0.2–0.5)	**<0.0001**	**0.0262**

*After the median values, interquartile ranges are shown in brackets.*

*PFIC patients compared to ASC patients. Significant differences are shown in bold. **Controls compared to ASC patients. Significant differences are shown in bold. ***Controls compared to PFIC patients. Significant differences are shown in bold.

*ASC, autoimmune sclerosing cholangitis; BA, bile acids; G, glycine; IQR, interquartile range; PFIC, progressive familial intrahepatic cholestasis; T, taurine.*

Equivalently to tBA, total MCA (tMCA) were significantly higher in ASC patients (median: 0.59 μmol/L, IQR: 0.16–1.00) and in PFIC patients (median: 7.39 μmol/L, IQR: 1.31–37.13) compared to age-matched controls (median: 0.05 μmol/L, IQR: 0.001–0.16, ASC *p* = 0.0006; PFIC *p* < 0.0001). TMCA levels in ASC and PFIC patients did not differ significantly (*p* = 0.1807) (Figure 1E).

Only in PFIC patients, ATX antigen levels were significantly higher (median: 1,650 ng/ml, IQR: 776.9–3,742) than in age-matched controls (median: 315.9 ng/ml, IQR: 251.1–417.2; PFIC *p* = 0.0003). We could neither find a statistically significant difference of ATX antigen levels between ASC patients (median: 435.9 ng/ml, IQR: 315.8–662.8) and age-matched controls (*p* = 0.0721) not between ASC- and PFIC patients (*p* = 0.0713) (Figure 1F).

Due to the small study group of PFIC patients, only ASC patient were stratified according to presence/absence of pruritus in the following.

### Total Bile Acid Levels in Pediatric Autoimmune Sclerosing Cholangitis Patients

In ASC patients with pruritus, tBA levels were slightly but significantly higher (median: 76.5 μmol/L, IQR: 54.7–205.1) compared to ASC patients without pruritus (median: 24.3 μmol/L, IQR: 16.2–80.8; *p* < 0.0408) (Figure 2A). The T-conjugated BA were substantially higher in ASC patients with pruritus (median: 16.4 μmol/L, IQR: 8.9–41.4) than in ASC patients without pruritus (median: 1.3 μmol/L, IQR: 0.8–4.9; *p* = 0.0023) (Figure 2B). Likewise, G-conjugated BA were significantly increased in ASC patients with pruritus (median: 57.9 μmol/L, IQR: 44.6–148.8) compared to ASC patients without pruritus (median: 14.3 μmol/L, IQR: 9.2–39.3; *p* = 0.0200) (Figure 2C). There was no statistically significant difference in unconjugated BA between ASC patients with and without pruritus (*p* = 0.4442) (Figure 2D).

### Bile Acid Profiles in Pediatric Autoimmune Sclerosing Cholangitis Patients

The BA profiles of ASC patients with and without pruritus and healthy age-matched controls are shown in Figure 3A. The relative contribution of unconjugated BA levels to the total BA pool was lower in pediatric ASC patients with pruritus compared to ASC patients without pruritus including a substantially smaller fraction of UDCA. In contrast, T-conjugated BA made up a larger proportion in ASC patients with pruritus compared to patients without pruritus. G-conjugated BA predominated in all study groups. The G-conjugated primary BA, CDCA, and CA, and the secondary BA, UDCA, predominated in both, ASC patients with and without pruritus.

### Muricholic Bile Acid Levels in Pediatric Autoimmune Sclerosing Cholangitis Patients

TMCA levels in ASC patients with pruritus were substantially higher (median: 1.15 μmol/L, IQR: 0.77–2.44) compared to ASC patients without pruritus (median: 0.30 μmol/L, IQR: 0.13–0.64, *p* = 0.0033) (Figure 2E).

### Muricholic Acid Profiles in Pediatric Autoimmune Sclerosing Cholangitis Patients

The MCA profiles of ASC patients with and without pruritus are shown in Figure 3B. Generally, in ASC patients with pruritus, the MCA pool showed a larger variance. Same as in “typical” BA profiles, unconjugated MCA were lower in ASC patients with pruritus with a predominance of HDCA. In both, ASC patients with and without pruritus, T-conjugated MCA constituted a larger fraction than in “typical” BA profiles. Especially TOMCA was detectable in equal amounts in both groups. In ASC patients with pruritus, TGMCA and TAMCA and GGMCA were the most frequent MCA. In ASC patients without pruritus, the relative contribution of G-conjugated MCA to the total MCA pool was larger than in ASC patients without pruritus with a predominance of GAMCA and GGMCA.

### Autotaxin Antigen Levels in Pediatric Autoimmune Sclerosing Cholangitis Patients

ATX antigen levels in ASC patients with pruritus, (median: 665.8 ng/ml, IQR: 357.8–1,203) and in ASC patients without pruritus (median: 391.0 ng/ml, IQR: 283.2–485.6) were not significantly different (*p* = 0.1061) (Figure 2F).

### Correlation of Autotaxin, Visual Analog Scale of Pruritus, Bile Acids and Muricholic Acids in Pediatric Autoimmune Sclerosing Cholangitis Patients

ATX antigen levels correlated significantly with serum tBA levels in pediatric ASC patients with pruritus (Spearman *r* = 1, *p* = 0.0028) but not with tBA levels in ASC patients without pruritus (Spearman *r* = 0.53, *p* = 0.079). Moreover, ATX antigen levels correlated significantly with T- and G-conjugated BA in ASC patients with pruritus (T: *r* = 0.8857, *p* = 0.0333; G: *r* = 0.9429, *p* = 0.0167), and without pruritus (T: *r* = 0.7231, *p* = 0.0047; G: *r* = 0.5956, *p* = 0.0274).

Interestingly, ATX antigen levels did not correlate significantly with tMCA levels in pediatric ASC patients with pruritus (Spearman *r* = 0.37; *p* = 0.4972) but with tMCA levels in patients without pruritus (Spearman *r* = 0.87; *p* = 0.0001).

PVAS values did not correlate with tBA levels (Spearman *r* = 0.05, *p* = 0.9316), tMCA levels (Spearman *r* = 0.62, *p* = 0.3787) or ATX values (Spearman *r* = 0.38, *p* = 0.4722) in pediatric patients with ASC and pruritus. Moreover, PVAS did not correlate with T-conjugated BA (*r* = 0.4348, *p* = 0.3778), G conjugated BA (*r* = 0.4928, *p* = 0.3333), or unconjugated BA (*r* = 0.3189, *p* = 0.5556) in ASC patients with pruritus.

## Discussion

Various CCLD such as PFIC or ASC are frequently associated with chronic pruritus in pediatric but also in adult patients. However, the exact etiology is unknown and itch intensity does not correlate with CCLD severity or common biochemical indices of liver disease (3, 4). Furthermore, the treatment of chronic cholestatic pruritus represents a clinical challenge: Rifampicin is a PXR-agonist, which increases the metabolism and secretion of pruritogenic substances and is often used as first-line therapy in children but not consistently effective (32, 33). Albeit widely prescribed, the use of UDCA in CCLD is highly controversial as it has not been shown to ameliorate pruritus in PBC or PSC (34–36). In cholestatic states in which BA secretion in bile and intestine is low, such as PFIC or biliary atresia, anion exchanger resins as cholestyramine have low efficacy (37, 38). Medications modulating central pruritus-transmission include opioid antagonists, like naltrexone or naloxone, and SSRI. An improvement of pruritus has been suggested in small pediatric series only (39–41). Novel therapeutic options for pruritus in CCLD are ASBT-inhibitors, such as odevixibat, which inhibit ileal BA reabsorption. In children, improvement of pruritus severity was reported with PFIC and various other CCLD (42, 43). Another targeted therapeutic option is the chaperone 4-phenylbutyrate, which has been tested in children with PFIC2. Treatment with 4-phenylbutyrate was associated with re-expression of BSEP and improvement of pruritus (44). As most available therapeutic options are largely empiric new insights into the pathophysiology of chronic pruritus in cholestasis are indispensable in pediatric and adult populations.

In our study, we found significantly higher tBA levels in pediatric PFIC and ASC patients independently of existing pruritus compared to healthy age-matched controls which was expected since it is characteristic for PFIC/ASC pathophysiology. Especially the G- and T-conjugated BA were significantly higher in both, PFIC and ASC patients.

However, in ASC patients with pruritus, we found only slightly higher tBA levels compared to ASC patients without pruritus and no correlation between tBA and PVAS. This is in line with other studies investigating adult and pediatric CCLD patients with pruritus that could not demonstrate a correlation between pruritus severity and tBA levels or single BA levels, respectively (5, 6). Kremer et al. reported a correlation of tBA and PVAS in pediatric CCLD patients with pruritus (26), which we did not observe. However, they included children suffering from Alagille syndrome, extrahepatic biliary atresia, neonatal sclerosing cholangitis, and PFIC but not ASC patients.

On the other hand, we found an altered serum BA profile associated with pruritus. T-conjugated BA were significantly higher and the fraction of unconjugated BA in the total BA pool was lower in ASC patients with pruritus compared to ASC patients without pruritus. A higher degree of conjugation may be caused by upregulation of BACS and BAT secondary to increased FXR stimulation (23, 24). An alternative explanation would be decreased deconjugation by gut microbiomes with reduced bile salt hydroxylase. We could not test this hypothesis, since stool samples were not available for microbiome analysis. Based on our findings, a clinical differentiation of ASC with and without pruritus seems useful in children and adolescents as BA profiles were not comparable despite the same underlying disease. Furthermore, the higher concentration of T-conjugated BA is an interesting finding because differing ratios of G- vs. T-conjugates have been reported in PBC vs. PSC patients (45) and may be associated with a varying autoimmune component. The potential role of T-conjugated BA in the development of pruritus remains to be investigated.

Same as with human tBA, tMCA were significantly increased in PFIC and ASC patients compared to controls. TMCA levels were highest in our PFIC patients with rifampicin-therapy, which is known to induce C6-hydroxylation. Pediatric ASC patients with pruritus had significantly higher tMCA levels compared to ASC patients without pruritus. Furthermore, we found a distinctly different MCA profile in ASC patients with pruritus compared to ASC patients without pruritus featuring less heterogenicity.

Besides usual BA, the existence of MCA, a group of tri-hydroxylated BA mainly found in mice, is reported in umbilical cord blood and amniotic fluid from neonates (14, 15). Interestingly, various MCA have been identified in patients with cholestatic liver diseases (46), which suggests the existence of some altered pathways of BA metabolism in fetal, neonatal, and cholestatic liver. Alternatively, MCA may constitute a minuscule fraction of the normal human BA pool becoming increasingly detectable with accumulation during cholestasis. MCA are also influencing FXR: AMCA is considered to have antagonistic effects (47). Interestingly, T-conjugation (TAMCA) predominated in our ASC patients with pruritus whereas G-conjugation (GAMCA) was prevailing in ASC patients without pruritus. If there is a difference in the antagonistic potential of FXR between TAMCA and GAMCA, however, needs further investigation.

TOMCA was previously found to be significantly increased in preterm neonates with early onset sepsis compared to preterm controls and was therefore considered as potential biomarker in septic neonates (48). However, TOMCA levels were comparable in our study cohort and could therefore not distinguish between ASC patients with or without pruritus. Therefore, the diagnostic potential of TOMCA and MCA in general needs further investigation by ongoing studies in pediatric CCLD patients with pruritus. On the one hand, UDCA and HDCA levels were substantially lower in our patients with ASC patients with pruritus, compared to ASC patients without pruritus. Hence, a potential role of UDCA and HDCA as possible biomarkers for pruritus in pediatric ASC patients are conceivable. On the other hand, reduction of UDCA and HDCA might be a general sign of aggravated cholestasis and upregulation of conjugating enzymes (BAC, BAT) secondary to FXR overstimulation (23, 24).

In our study, ATX antigen levels were significantly elevated in PFIC patients but not in ASC patients compared to control subjects. In ASC patients, ATX antigen levels were neither significantly increased compared to age-matched controls, nor did they significantly differ in dependence of presence/absence of pruritus.

We measured ATX antigen, as it has been shown to play a role in cholestatic pruritus by generating LPA, a potent neuronal activator, which is considered as potential pruritogen (49). Kremer et al. found an increased ATX activity in serum of cholestatic adult patients (intrahepatic cholestasis in pregnancy, primary biliary cirrhosis) with pruritus compared to cholestatic adult patients without pruritus. Furthermore, ATX activity correlated with the intensity of pruritus (27, 50). Besides adult patients, Kremer et al. also described significantly increased ATX levels in pediatric CCLD patients with pruritus (Alagille syndrome, extrahepatic biliary atresia, neonatal sclerosing cholangitis, PFIC) compared to children with BA synthesis defects, in which pruritus is typically not observed and tBA are low (26). Same as in the study of Kremer et al. ATX levels correlated with tBA- but not with tMCA levels in our pediatric ASC patients with and without pruritus (26). Based on our findings, ATX antigen levels seem to have little diagnostic or prognostic meaning in ASC patients different to other CCLD such as Alagille syndrome or PFIC. Furthermore, an increased ATX activity alone seems not to be causal for pruritus genesis in ASC patients.

To the best of our knowledge this is the first study representing a distinct different BA- and MCA profile and enhanced tMCA levels in ASC patients with pruritus compared to ASC patients without pruritus. The novelty of this paper is the investigation of MCA, which, in contrast to “typical” BA show a substantial surge in ASC patients with pruritus and an almost complete differentiation of murine BA levels between the groups. This surge of murine BA is a novel phenotypic observation associated with pruritus that may allow a better classification of ASC patients.

We acknowledge that the representativeness of our result may be limited due to a small number of analyzed patients per study group and need therefore further investigation by ongoing studies. However, we think that our findings have a clinical impact as they give new insights of the pathophysiological course of ASC patients and indicate that the extent of ATX activity alone is not a trigger of pruritus in pediatric ASC patients. Moreover, we acknowledge that the serum samples of our study groups were taken in a non-fasting state which was due to ethical reasons in the pediatric age group. For that reason, alterations compared to fasting states cannot be excluded entirely between our PFIC/ASC patients with and without pruritus. Furthermore, ongoing *in-vitro* studies investigating the cellular source of ATX and the influence of BA on ATX secretion need to exclude confounding factors.

## Conclusion

To date, data in the pediatric population suffering pruritus in cholestasis are sparse. To our knowledge, this is the first study in pediatric patients investigating MCA levels and BA/MCA profiles in children with PFIC and ASC with and without pruritus.

In summary, we observed slightly elevated serum tBA and significantly elevated tMCA levels in pediatric ASC patients with pruritus compared to pediatric ASC patients without pruritus and age-matched controls. Particularly due to increased T conjugated BA/MCA, pediatric ASC patients with pruritus show a distinctly different BA- and MCA profile than ASC patients without pruritus despite the same underlying disease. Different to PFIC patients, ATX antigen levels were not significantly increased in ASC patients compared to controls. Moreover, we could not find significant differences in ATX antigen levels between ASC patients with or without pruritus indicating that ATX is not the sole cause for pruritus genesis in ASC patients contrasting other CCLD such as Alagille syndrome or PFIC. However, our results need further investigation by ongoing *in-vitro* studies investigating the cellular source of ATX. Even though the exact pathophysiology of pruritus in cholestasis remains unknown, a clinical differentiation of ASC with and without pruritus seems useful in children and adolescents.

## Data Availability Statement

The original contributions presented in the study are included in the article/Supplementary Material, further inquiries can be directed to the corresponding author/s.

## Ethics Statement

The studies involving human participants were reviewed and approved by the Hungarian Ethics Committee (43477-/2018/EKU) Austrian Ethics Committee (31–337 ex 18/19). Written informed consent to participate in this study was provided by the participants’ legal guardian/next of kin.

## Author Contributions

KM and JJ conceived the initial idea and wrote the manuscript with the support of AS. DS and AD provided demographic data and serum samples and our pediatric study group with chronic cholestatic liver diseases. KM, SP, and TS planned and performed the measurements. KM analyzed the data and constructed figures and tables. All authors provided critical feedback and helped shape the manuscript.

## Conflict of Interest

The authors declare that the research was conducted in the absence of any commercial or financial relationships that could be construed as a potential conflict of interest.

## Publisher’s Note

All claims expressed in this article are solely those of the authors and do not necessarily represent those of their affiliated organizations, or those of the publisher, the editors and the reviewers. Any product that may be evaluated in this article, or claim that may be made by its manufacturer, is not guaranteed or endorsed by the publisher.

## Footnotes

1
https://www.drks.de/drks_web/

## Supplementary Material

Supplementary Figure 1 Overview of groups and number of all pediatric participants. ASC, autoimmune sclerosing cholangitis; PFIC, progressive familial intrahepatic cholestasis.
