# Supplementary figures and images for: The Covert Surge: Murine Bile Acid Levels Are Associated With Pruritus in Pediatric Autoimmune Sclerosing Cholangitis
Source: Front Pediatr. 2022 May 11;10:903360. doi: 10.3389/fped.2022.903360 (PMC9130722; doi:10.3389/fped.2022.903360)

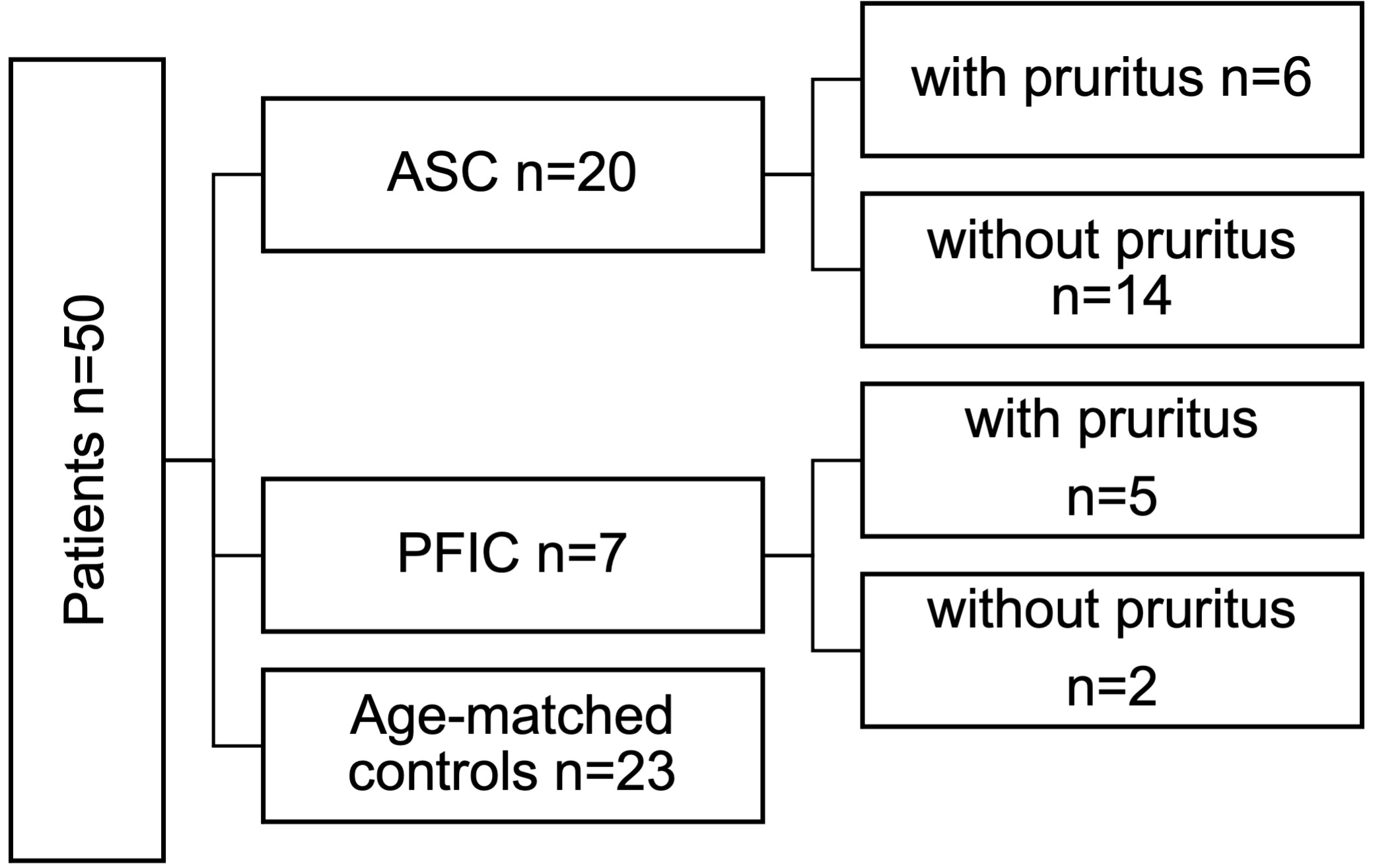

Supplement: Supplementary Figure 1 — Overview of groups and number of all pediatric participants. ASC, autoimmune sclerosing cholangitis; PFIC, progressive familial intrahepatic cholestasis. [file Image_1.JPEG]
